# Frontiers in Bone Metabolism and Disorder in Chronic Kidney Disease

**DOI:** 10.3390/metabo13101034

**Published:** 2023-09-26

**Authors:** Maria L. Mace, Ewa Lewin

**Affiliations:** 1 Department of Nephrology, Rigshospitalet, University of Copenhagen, 2100 Copenhagen, Denmark; 2 Department of Nephrology, Herlev Hospital, University of Copenhagen, 2100 Copenhagen, Denmark; lewin@dadlnet.dk

Chronic Kidney Disease (CKD) is a progressive condition that affects 10–15% of the adult population, a prevalence expected to increase worldwide [1]. CKD has deleterious systemic effects on the viability and function of several organs, namely the cardio-vascular system, bone metabolism, the immune system, muscle strength, energy balance, fertility, and cognitive functions. Of all these many complications to CKD, the mineral and bone disorder (CKD-MBD) stands out as the most serious one affecting morbidity and mortality [2].

The disturbances in the mineral homeostasis start at the early stages of CKD progression with a reduction in α-Klotho and an increase in fibroblast growth factor 23 (FGF23), this is followed by a downregulation of the active vitamin D metabolite 1,25 dihydroxy vitamin D_3_ (1,25 vitamin D). Subsequently, plasma levels of parathyroid hormone (PTH) increase, and so does glandular hyperplasia due to low calcium, phosphate retention, skeletal resistance to PTH signaling and abnormalities in the regulatory feedback loops between FGF23 and 1,25 vitamin D. These changes in minerals and their regulatory hormones are accompanied by changes in bone morphology, bone density and remodeling activity as well as development of soft tissue calcification especially in the arteries and heart valves [3,4]. Current treatment therapies are aimed at vitamin D insufficiency, hyperphosphatemia and the secondary hyperparathyroidism in the later stages of CKD. However, CKD patients continue to suffer from high cardiovascular morbidity and mortality as well as increased fracture risk, calling for improved treatment of this serious complication to CKD. The extensive research into CKD-MBD has improved the understanding of the complex pathological mechanisms with recent important findings. This Special Issue provides valuable new insights into the complex CKD-MBD syndrome.

Phosphate retention and hyperphosphatemia are still seen as one of the major detrimental factors in CKD-MBD. In line with decreasing kidney function, this essential mineral becomes a uremic toxin. Plasma levels of phosphate are kept within normal range until later stages of CKD due to the increase in phosphaturic hormones FGF23 and PTH. At this stage of CKD, many patients already suffer from vascular calcification. Treatment with phosphate binders do decrease plasma levels of phosphate; however, this treatment has not been shown to improve cardiovascular outcomes in CKD patients. Evaluating plasma phosphate in CKD patients is further complicated by the recently demonstrated disturbed circadian rhythm in phosphate and PTH levels [5,6]. CKD affects the internal parathyroid circadian clock, which is another factor contributing to parathyroid hyperplasia in CKD [7]. The parathyroid gland expresses the calcium-sensing receptor (CaSR), which serves as the main regulator of PTH secretion. Interestingly, it was recently shown that the CaSR has a phosphate-binding element, which, upon binding to phosphate, alters the configuration of the receptor to its inactive state and hereby triggers PTH secretion [8]. Minor deviations in plasma ionized calcium are rapidly corrected by influx or efflux of calcium on the bone surface. In contrast, plasma phosphate fluctuates more with a slower response to hyperphosphatemia [9,10]. Although the hormonal regulation of extracellular phosphate is increasingly characterized, the tissue and intracellular phosphate handling is poorly understood. A recent study reported the exiting findings of a new intracellular organelle, which stores phosphate and hereby regulates cytosolic phosphate concentration [11].

Central to the treatment of CKD-MBD is the management of the secondary hyperparathyroidism. Several advances have been achieved in understanding PTH regulation and parathyroid gland proliferation in CKD; in-depth understanding of these regulatory pathways on a molecular level may lead the way for future research and new treatment. Increased PTH mRNA stability is one of the main mechanisms that increases PTH gene expression in secondary hyperparathyroidism. The Pin1 enzyme holds a key role in inducing PTH mRNA decay, and so decreased Pin1 activity results in increased PTH levels [12]. The role of microRNAs (miRNAs) in regulating gene expression is a research focus in many fields. Recently, the miRNA profile of the parathyroid gland from end-stage kidney patients have been studied, showing substantial alterations in the expression levels of miRNAs. Also, the study identified a subgroup of miRNAs that may be involved in secondary hyperparathyroidism [13]. In this Special Issue, Hassan et al. combine the substantial evidence of the molecular mechanism in PTH regulation and secretion, including secondary hyperparathyroidism in CKD [14]. The disturbed vitamin D metabolism in CKD has been known for many years, but the beneficial effect of vitamin D supplements in CKD is still uncertain [15]. Ureña Torres et al. overview the current evidence on the use of vitamin D supplement and bone fragility in CKD. Moreover, they present an expert opinion on ways to target this complication in CKD patients in this Special Issue [16].

The disturbed α-Klotho/FGF23 system has been demonstrated to play an early and detrimental role in CKD-MBD. Several new pieces of evidence have expanded our knowledge of the complex regulation of FGF23. Recent studies have elucidated that phosphate regulates FGF23 in bone. The phosphate molecule binds the receptors to the inorganic phosphate transporter 1 (Pit1) and the FGF23’s receptor FGFR1, affecting the receptors’ activity and thereby increasing FGF23 secretion [17,18,19]. Still, FGF23 secretion seems not to respond rapidly to acute changes in plasma levels of either phosphate or calcium, but more to the intake of these minerals [9,20,21,22]. Calciprotein particles (CPPs) are circulating soluble nanoparticles consisting of the Fetuin A protein and the precipitates of calcium and phosphate. These molecules protect the organism from the spontaneous crystal formation from the supersaturated concentrations of calcium and phosphate in the extracellular fluid [23]. CPPs have been a focus for CKD-MBD research, especially the shift from the amorphous primary CPPs to the larger and more toxic secondary CPPs [24]. This defence system seems to be linked to FGF23 function, as the primary CPPs stimulate FGF23 in bone and thereby regulate phosphate homeostasis [25]. In addition to the classical endocrine feedback loops between FGF23, PTH and 1,25 vitamin D, several factors regulate FGF23 levels. Still, the kidney holds a key role in regulating plasma levels of FGF23 by renal extraction of the hormone [9]. CKD causes a decrease in the renal clearance of FGF23, which, together with multiple stimulators of FGF23 expression, all lead to the extreme plasma levels of FGF23 in CKD patients. These high FGF23 levels are thought to have negative off-target effects by activating FGF receptors in other organ systems. The balance between an essential phosphaturic hormone and potential uremic toxin challenges the potential treatment to target the FGF23 increase. In this Special Issue, Vervloet, M. provides a comprehensive review of the complex regulation of FGF23, highlighting potential treatment targets for future research in CKD-MBD. Furthermore, the review provides a nexus between dysregulated FGF23 and clinical manifestations [19].

In recent years, research has suggested that other factors and signal pathways seem to be involved in the pathogenesis of renal osteodystrophy. The anabolic Wnt pathway in bone is disturbed at the early stages of CKD with an increased expression of the Wnt inhibitor sclerostin [26,27]. Plasma levels of sclerostin are increased in CKD patients. In this Special Issue, Cejka, D. analyzes the current understanding of sclerostin as a biomarker and a potential treatment target in CKD patients [28].

Cardiovascular disease is still the most serious complication to CKD causing increased morbidity and mortality. The majority of CKD patients suffer from both arterial lamina intima and lamina media calcification, although the latter is characteristic for vascular disease in CKD for which there is no specific treatment. The arterial media calcification runs longitudinal through the vessels impairing the elastic properties as well as the dynamics of vessel’s tonus. The change in the vascular bed has a detrimental effect on the heart resulting in hypertrophy and heart failure. Calcification of the medial layer of the arteries is characterized by the shift in phenotype of the vascular smooth muscle cell (VSMC) to a bone-like secretory cell. Other important cell types involved in pathogenesis are the endothelial cell, which may undergo endothelial to mesenchymal transition (EndMT), and stem cells (Gli1 + cells) from the adventitia, which may migrate and differentiate into bone-like cells [29]. The vascular calcification process is very similar to bone formation and mineralization. Therefore, potential treatments of vascular calcification most likely have an adverse effect on bone metabolism [30,31]. One promising way to overcome this obstacle is to use nanomedicine (nanoparticle-based drug delivery systems) with the ability to conjugate specific proteins on the surface of the nanoparticle. In a recent study, an elastin antibody was conjugated to albumin nanoparticle loaded with a calcium-chelating agent in order to target the compound to the vasculature, where it inhibited the development and progression of vascular calcification in CKD rats. The treatment did not affect bone metabolism [32]. In this Special Issue, Van den Branden et al. provide a substantial insight into the pathophysiology of vascular calcification and present the latest research trying to target the media calcification, elucidating potential future treatments in a clinical setting [31].

The multiple systemic effects of CKD have traditionally been explained by a reduction in glomerular filtration, resulting in an accumulation of waste products and uremic toxins along with the low grade of chronic inflammation and hormonal disturbances. Nevertheless, a new hypothesis of CKD-MBD was proposed by Hruska et al. More specifically, disease processes in the kidney activate development programs normally involved in nephrogenesis. These development programs are involved in renal repair or fibrosis processes. Yet, factors are secreted from the kidney, resulting in negative effects on the vasculature and bone in CKD [33]. The interesting paradigm of disturbed system biology in CKD is adding to the complexity of kidney disease.

Our group has studied vascular calcification in CKD models and found significant changes in the transcriptome of the calcified artery. Interestingly, the calcified artery expressed factors normally used for cell communication in the microcirculation in the bone (e.g., angiokines and osteokines). We found an overall increase in endothelial-derived factors known to stimulate bone formation [34]. Therefore, we speculate whether the arterial endothelial cell also makes a phenotypic shift to a vascular endothelial cell found in bone. This de-differentiated endothelial cell communicates with the vascular bone-like cell and thereby stimulates vascular bone formation in CKD. We propose this new hypothesis of osteomimicry. As established vascular calcification is not reversed by transplantation, it is highly important to focus on prevention [35]. Identifying these early signals in the pathogenesis of vascular calcification may elucidate a potential treatment target. For several decades, an association between low bone mineral density and the presence of vascular calcification in normal aging and disease has been known. Still, the mechanisms leading to this “calcification paradox” have been poorly understood [36]. CKD-MBD research have focused on the disturbed mineral and bone metabolism in triggering and exacerbating the severe vascular calcification seen in CKD patients. However, our group has identified a detrimental role of the calcified vasculature in CKD-MBD. More specifically, the calcified arteries secrete several signal molecules related to Wnt and TGF-β pathways; these factors have direct negative effects on bone metabolism. This leads to a negative spiral of demineralization of bone and mineralization of the vasculature, a pathological vascular–bone tissue crosstalk in CKD-MBD [37]. This new paradigm improves the understanding of the link between bone demineralization and vascular mineralization, which has been observed for years. In this Special Issue, we discuss the new insights into the crosstalk between the vasculature and bone tissue in CKD-MBD [38].

In conclusion, this Special Issue sums up the latest research in the field of CKD-MBD and provides novel insights into the complex disorder. The new understanding and novel concepts may lead future research and improvement of the treatment of CKD-MBD.
